# Abundance of Secreted Proteins of *Trichoderma reesei* Is Regulated by Light of Different Intensities

**DOI:** 10.3389/fmicb.2017.02586

**Published:** 2017-12-22

**Authors:** Eva Stappler, Jonathan D. Walton, Sabrina Beier, Monika Schmoll

**Affiliations:** ^1^Center for Health and Bioresources, AIT Austrian Institute of Technology GmbH, Tulln, Austria; ^2^MSU-DOE Plant Research Laboratory, Department of Plant Biology, Michigan State University, East Lansing, MI, United States

**Keywords:** *Trichoderma reesei*, *Hypocrea jecorina*, cellulase gene expression, secretion, light tolerance, protease

## Abstract

In *Trichoderma reesei* light is an important factor in the regulation of glycoside hydrolase gene expression. We therefore investigated the influence of different light intensities on cellulase activity and protein secretion. Differentially secreted proteins in light and darkness as identified by mass spectrometry included members of different glycoside hydrolase families, such as CBH1, Cel3A, Cel61B, XYN2, and XYN4. Several of the associated genes showed light-dependent regulation on the transcript level. Deletion of the photoreceptor genes *blr1* and *blr2* resulted in a diminished difference of protein abundance between light and darkness. The amount of secreted proteins including that of the major exo-acting beta-1,4-glucanases CBH1 and CBH2 was generally lower in light-grown cultures than in darkness. In contrast, *cbh1* transcript levels increased with increasing light intensity from 700 to 2,000 lux but dopped at high light intensity (5,000 lux). In the photoreceptor mutants Δ*blr1* and Δ*blr2* cellulase activity in light was reduced compared to activity in darkness, showing a discrepancy between transcript levels and secreted cellulase activity. Furthermore, evaluation of different light sensitivities revealed an increased light tolerance with respect to cellulase expression of QM9414 compared to its parental strain QM6a. Investigation of one of the differentially expressed proteins between light and darkness, CLF1, revealed its function as a factor involved in regulation of secreted protease activity. *T. reesei* secretes a different set of proteins in light compared to darkness, this difference being mainly due to the function of the major known photoreceptors. Moreover, cellulase regulation is adjusted to light intensity and improved light tolerance was correlated with increased cellulase production. Our findings further support the hypothesis of a light intensity dependent post-transcriptional regulation of cellulase gene expression in *T. reesei*.

## Introduction

The filamentous ascomycete *Trichoderma reesei* (syn. *Hypocrea jecorina*) is an important producer of industrial enzymes, especially cellulases for conversion of cellulosic biomass (Bischof et al., 2016; Paloheimo et al., 2016; Schmoll et al., 2016). Especially for the production of biofuels from cellulosic waste material the enzymes of *T. reesei* are very important (Kumar et al., 2008). Additionally *T. reesei* is a frequently used host for the production of heterologous proteins (Nevalainen and Peterson, 2014; Singh et al., 2015). For *T. reesei*, glycoside hydrolase gene expression is regulated by light at the transcriptional level (Schmoll et al., 2005; Tisch et al., 2011b; Tisch and Schmoll, 2013).

Light is an important environmental cue for most living organisms (Dunlap and Loros, 2017). Changes in light conditions, as a result of diurnal cycles or of growth on the surface compared to within a substrate, lead to considerably altered physiological processes in fungi. Light influences diverse functions like sexual development, conidiation, intracellular levels of ATP and cyclic adenosine monophosphate (cAMP), and many metabolic processes (Corrochano, 2007; Rodriguez-Romero et al., 2010; Tisch and Schmoll, 2010; Schmoll, 2011). In ascomycetes, the perception of light signals is largely conserved, with detection of blue, red and green light depending on the species (Idnurm and Heitman, 2005). Thereby, two GATA-type transcription factors containing PER Arnt Sim (PAS) domains are crucial for light perception (Schafmeier and Diernfellner, 2011). They act on a flat hierarchy, targeting transcriptional regulators, which in turn act on downstream pathways (Smith et al., 2010). The light perception machinery of *T. reesei* consists of BLR1, BLR2 and ENV1, which all contain PAS domains and have functions predominantly in light, but also in darkness (Schuster et al., 2007; Schmoll et al., 2010; Tisch and Schmoll, 2013; Schmoll, in press). BLR1 and BLR2 (blue light regulator 1 and 2) are homologs of the *N. crassa* photoreceptors White Collar-1 (WC-1) and White Collar-2 (WC-2), two GATA zinc-finger transcription factors that together form the White Collar Complex (WCC) to transfer signals to their target genes (Brunner and Kaldi, 2008; Chen et al., 2010). BLR1 and BLR2 regulate growth in light conditions and modulate cellulase gene transcription (Castellanos et al., 2010; Gyalai-Korpos et al., 2010; Schmoll et al., 2010). Although they are expected to act as a complex, BLR1 and BLR2 as well as their homologs in *N. crassa* also have individual functions (Schmoll et al., 2012; Tisch and Schmoll, 2013).

A third photoreceptor in *N. crassa* is VIVID, a PAS/LOV domain protein that is involved in detecting changes in light intensity as well as in adaptation to constant light (Heintzen et al., 2001; Hunt et al., 2010). VIVID is assumed to sense the difference between changes in light intensity during the day and night (moonlight) and is essential for photoadaptation, during which it binds to the WCC and acts as a universal brake for photoresponses (Chen et al., 2009; Malzahn et al., 2010). Its ortholog in *T. reesei*, ENV1 (Schmoll, in press), is not a functional homolog, although it shares similar functions (Schmoll et al., 2005; Castellanos et al., 2010). Particularly, deletion phenotypes are different, while the photoreceptors BLR1 and BLR2 are essential for *env1* induction as in *N. crassa* (Schmoll et al., 2005; Castellanos et al., 2010). One further important difference is in the cysteine residue at position 96, which integrates oxidative stress signaling with light response in Hypocreales (Lokhandwala et al., 2015). ENV1 is necessary for normal growth in light and for photoadaptation, as well as for responding to different light intensities (Schmoll et al., 2005; Schuster et al., 2007; Castellanos et al., 2010) and has functions in sexual development (Seibel et al., 2012) and regulation of the heterotrimeric G-protein pathway (Tisch et al., 2011a).

The proteome of *T. reesei* has been analyzed previously under different conditions. The types and abundance of secreted and intracellular proteins strongly depend on the carbon source for growth (Adav et al., 2012; Jun et al., 2013; Peciulyte et al., 2014). The most efficient protein secretion rate occurs at low specific growth rates (Pakula et al., 2005; Arvas et al., 2011). Analysis of proteome-wide phosphorylation revealed a complex signaling network for cellulase induction that includes components of carbon sensing, osmoregulation and light signaling (Nguyen et al., 2016).

Due to the altered regulation of cellulase gene expression and phenotypic characteristics in light in *T. reesei*, we became interested whether the light intensity is crucial for regulation. Here we studied the secreted proteins of *T. reesei* under four different light conditions, ranging from 700 to 5,000 lux, during growth on cellulose. Traditionally, besides the original isolate QM6a, different strains of *T. reesei* are used for functional genomics analysis (Guangtao et al., 2009; Schuster et al., 2012a), which caused the question whether the genetic background is relevant for light tolerance. Hence, we compared the effect of light and light sensitivity on both the ancestral isolate QM6a and the cellulase high producer QM9414, and also evaluated the influence of the photoreceptors BLR1, BLR2, and ENV1 on the secretome in light and dark. Analysis of one of the differentially regulated proteins revealed that it had a function in regulating protease activity in *T. reesei*.

## Materials and methods

### Fungal strains and culture conditions

*Trichoderma reesei* wild-type strain QM6a, its derivative QM9414 (ATCC 26921), QM9414Δ*blr1*, Δ*blr2*, and Δ*env1* (Castellanos et al., 2010), and QM6aΔ*ku80* were used throughout this study. Strains were maintained on 3% (w/v) malt extract-agar (malt extract: Merck, Darmstadt, Germany; agar-agar: Roth, Karlsruhe, Germany). For quantitative reverse transcription-PCR (qRT-PCR) analysis, biomass determination, SDS-PAGE, and secreted cellulase activity, *T. reesei* was grown in liquid culture in 100 ml Mandels-Andreotti minimal medium (Mandels and Andreotti, 1978) supplemented with 0.1% (w/v) peptone (Roth, Karlsruhe, Germany) and with 1% (w/v) microcrystalline cellulose (Alfa Aesar, Karlsruhe, Germany) as a carbon source for 72 h at 28°C on a rotary shaker (200 rpm). Strains were grown either in the presence of constant illumination (Osram L 18W/835; day light simulating wave length distribution) with different light intensities ranging from 700 to 5,000 lux or in constant darkness. In the latter case, cultures were harvested under red safety light (Fischer Photolamp 230V 15W^*^5F, Diez, Germany) using Miracloth filtration material (Calbiochem/Merck, Darmstadt, Germany) to separate the culture from the supernatant.

### Construction of *T. reesei* deletion strains

The deletion vector for TR_111915 was constructed by yeast recombination as described earlier (Schuster et al., 2012a) using primers pdel111915_5F 5′ GTAACGCCAGGGTTTTCCCAGTCACGACGTCTCTTGAAGCCATGAAAGC 3′ and pdel111915_5R 5′ ATCCACTTAACGTTACTGAAATCTCCAACGGAGGAGGTAGATTAAAGGC 3′ (956 bp fragment) for PCR amplification of the 5′ region and pdel111915_3F 5′ CTCCTTCAATATCATCTTCTGTCTCCGACGAATGTAAAGAGCTGGACAC 3′ and pdel111915_3R 5′ GCGGATAACAATTTCACACAGGAAACAGCACAGAATCCAGCATAATGGC 3′ (994 bp fragment) for the 3′ region and the hph deletion cassette described therein. The deletion cassette was PCR-amplified with primers pdel111915_5F and pdel111915_3R (3,455 bp fragment). Ten microgram of the purified fragment were used for protoplast transformation of QM6a Δ*ku80* as described (Gruber et al., 1990). Transformants were selected on plates containing 100 μg/ml hygromycin B (InvivoGen, USA). Deletion of the open reading frame was tested by PCR using primers binding within the deleted region (RT_111915_F 5′ GACATGAAGTGCGTCCCCGACA 3′ and RT_111915_R 5′ CCTTCGGACAAGCCAACCCCAT 3′; 253 bp fragment). No amplicon was detectable in the mutant strain confirming removal of the region to be deleted. Integration of the cassette at the correct location was confirmed by PCR using primers pdel111915_SC 5′ ACATGTGGCCAAGGGAAATCGC 3′, binding outside the deletion cassette and hph_SC_R 5′ GATGATGCAGCTTGGGCGCAG 3′, binding inside the marker gene (1,292 bp fragment).

### RNA isolation and cDNA synthesis

Extraction of total RNA was carried out as described (Tisch et al., 2011a) using the RNeasy Plant Mini Kit (Qiagen, Hilden, Germany). The RNA concentration was measured with a Nanodrop spectrophotometer. The quality of total RNA was evaluated by agarose gel electrophoresis and the RNA 6000 Nano Kit with the Agilent 2100 Bioanalyzer. The threshold for minimum quality was set to RIN > 9 (Tisch et al., 2011a). A 1 μg portion of each total RNA sample was treated with DNase I (Thermo Fisher, Waltham, MA, USA) and then reverse-transcribed using the RevertAID H minus first-strand cDNA synthesis kit (Thermo Fisher, Waltham, MA, USA) using oligo(dT)18 primers.

### Quantitative RT-PCR

Quantitative RT-PCR was performed as described (Tisch et al., 2011a). All reactions were performed on a CFX96 Real-Time system machine (Bio-Rad) with the GoTaq qPCR Master Mix (Promega, Madison, WI, USA) and primers for *cbh1* and *L6e* (reference gene) (Tisch et al., 2011a). At least two biological replicates were analyzed with three technical replicates. Data was analyzed with qbase+ (Biogazelle) and the CFX Maestro (Bio-Rad) software (statistics including ANOVA).

### Biomass determination

Biomass in the presence of insoluble cellulose was analyzed as described (Schuster et al., 2011). Due to the presence of insoluble cellulose, biomass could not be measured directly, but the protein content of the mycelium produced is analyzed reflecting biomass. Briefly, strains were grown in liquid medium with cellulose as the carbon source as described above. Mycelia were harvested by filtration, frozen in liquid nitrogen, and ground in pre-cooled grinding jars in a Retsch Mill MM301 (Retsch, Haan, Germany) for 30 s with an oscillation frequency of 30 Hz. The powder was suspended in 0.1 M NaOH. This suspension was sonicated three times for 30 s and incubated for 3 h at room temperature. Samples were centrifuged for 10 min at 3,220 × g, and the supernatants were transferred to new tubes. The protein concentration, which we use here as a measure of biomass, was measured by the Bradford Protein Assay (Bio-Rad, Hercules, USA) with bovine serum albumin (BSA) as standard according to the manufacturer's instructions. For each strain three biological replicates with two technical replicates were analyzed. Statistical analysis was done using PSPP 1.0.1 (version August 2017).

### Determination of CMCase activity in culture filtrates

Strains were grown in liquid medium as described above. Endo-1,4-β-D-glucanase activity in culture filtrates was assayed with azo-CM-cellulose (S-ACMC-L, Megazyme, Wicklow, Ireland). CMCase activity was determined using five biological replicates. To determine specific CMCase activity, measured activity was correlated to the amount of biomass as reflected by protein content of mycelia. Statistical analysis was done using PSPP 1.0.1 (version August 2017).

### Protein recovery from culture filtrates, SDS-PAGE, and western blotting

Aliquots of liquid culture filtrates were precipitated with 0.25 volume of 100% (w/v) trichloroacetic acid (Sigma-Aldrich, Germany), mixed well and incubated on ice for 30 min. After centrifugation for 30 min at 16,000 × g, the pellet was washed twice with acetone (Roth, Karlsruhe, Germany). The pellet was dried at room temperature and redissolved in 2x SDS-PAGE loading buffer. Before loading, samples were heated for 10 min at 99°C. SDS-PAGE, Coomassie staining, and Western blotting were performed according to standard protocols (Ausubel et al., 2007). Relative quantitation of coomassie stained bands was done using the Image Lab software (Bio-Rad, Hercules, CA, USA). For analysis of cellulases in culture filtrate, proteins were blotted on a nitrocellulose membrane (RPN303D, Amersham™ Hybond™-ECL, GE Healthcare, Little Chalfont, United Kingdom) by wet electroblotting. Antibodies against the major cellulase CBH1 and CBH2 (Mischak et al., 1989) and a horseradish peroxidase-conjugated anti-mouse IgG (W4021, Promega, Madison, WI) were used for detection. For visualization, Clarity Western ECL Substrate (170-5061, Bio-Rad, Hercules, CA, USA) was used. Photographs were taken with the ChemiDoc (Bio-Rad, Hercules, CA, USA).

### Experimental LC/MS/MS

Gel bands were digested in-gel according to (Shevchenko et al., 1996) with modifications. Briefly, gel bands were dehydrated using 100% acetonitrile and incubated with 10 mM dithiothreitol in 100 mM ammonium bicarbonate, pH 8, at 56°C for 45 min, dehydrated again and incubated in the dark with 50 mM iodoacetamide in 100 mM ammonium bicarbonate for 20 min. Gel bands were then washed with ammonium bicarbonate and dehydrated again. Sequencing grade modified trypsin was prepared to 0.01 μg/μL in 50 mM ammonium bicarbonate and ~50 μL of this was added to each gel band so that the gel was completely submerged. Bands were incubated at 37°C overnight. Peptides were extracted from the gel by water bath sonication in a solution of 60% acetonitrile/1 % TCA and vacuum dried to ~2 μL. Peptides were then re-suspended in 2% acetonitrile/0.1% trifluoroacetic acid to 20 μL. From this, 10 μL were injected by a Waters nanoAcquity Sample Manager (www.waters.com) and loaded for 5 min onto a Waters Symmetry C18 peptide trap (5 μm, 180 μm × 20 mm) at 4 μL/min in 5% acetonitrile/0.1% formic acid. The bound peptides were eluted onto a Waters BH130 C18 column (1.7 μm, 100 μm × 150 mm) and eluted over 16 min with a gradient of 5–30% B in 8 min, ramped up to 90% B at 9 min and held for 1 min, then dropped back to 5% B at 10.1 min using a Waters nanoAcquity UPLC. Buffer A was 99.9% water/0.1% formic acid and buffer B was 99.9% acetonitrile/0.1% formic acid). Flow rate was 1 μL/min.

Eluted peptides were sprayed into a ThermoFisher LTQ Linear Ion trap mass spectrometer outfitted with a MICHROM Bioresources ADVANCE nano-spray source. The top five ions in each survey scan were subjected to data-dependent zoom scans followed by low energy collision induced dissociation (CID) and the resulting MS/MS spectra were converted to peak lists in Mascot Distiller, v2.4.3.3 (www.matrixscience.com) using the default LTQ instrument parameters. Peak lists were searched against all sequences available in the *T. reesei* protein database (downloaded from the Joint Genome Institute, http://www.jgi.doe.gov/) appended with common laboratory contaminants (downloaded from www.thegpm.org, cRAP project) using the Mascot searching algorithm, v2.4 (www.matrixscience.com). The Mascot output was then analyzed using Scaffold Q+S, v4.3.0 (www.proteomesoftware.com) to probabilistically validate protein identifications. Assignments validated in Scaffold with <1% false discovery rate were considered true. In addition, the minimum criteria for positive identification were at least two peptides and >95% probability as determined by Scaffold.

Mascot parameters for all databases were as follows: (1) up to two missed tryptic sites allowed, (2) fixed modification of carbamidomethyl cysteine, (3) variable modification of oxidation of methionine, (4) peptide tolerance of ±200 ppm, (5) MS/MS tolerance of 0.6 Da and (6) peptide charge state limited to +2 and +3.

Data are deposited in the MassIVE database under accession number MSV000081684.

### Protease activity

To determine protease activity, strains were grown on TSA plates (3 g/l tryptone (Merck, Darmstadt, Germany), 1 g/l soytone (Merck, Darmstadt, Germany), 1 g/l NaCl, 20 g/l agar) supplemented with 1.5% milk powder (Roth, Karlsruhe, Germany) at 28°C in constant darkness or constant light (1,800 lux). Protease activity was manifested by halo formation. The sizes of the cleared zones and hyphal extension were recorded and the ratio of the diameter of the halo to the diameter of the colony was calculated. Three biological and two technical replicates were analyzed.

## Results

### The secretome of *Trichoderma reesei* is influenced by light

**Figure 1:**
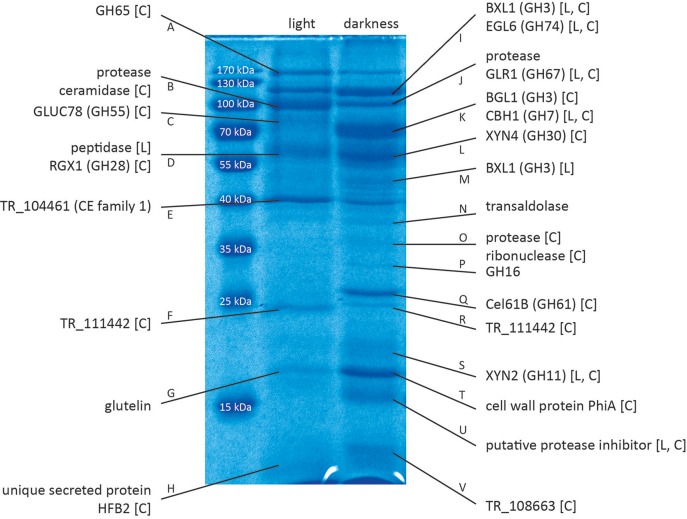
Proteins identified by mass spectrometry. Proteins were separated by SDS-PAGE and selected bands were excised and subjected to LC/MS/MS. Proteins were identified using the Mascot searching algorithm. [L] indicates regulation of the corresponding gene in response to light at the transcriptional level (Tisch et al., 2011b). [C] indicates regulation of the corresponding gene under cellulase-inducing conditions (Stappler et al., 2017). A figure with boxes around the proteins that were cut out for analysis is provided in Data sheet 1 (Figure S1). QM6a was grown on Mandels Andreotti minimal medium with 1% (w/v) cellulose as carbon source for 72 h in constant darkness or constant light (1,500 lux).

By SDS-PAGE we found considerable differences in the protein composition of culture filtrates of *T. reesei* during growth on cellulose in constant light or constant darkness (Figure 1, Figure S1 in Data sheet 1). This is consistent with the reported differences in the transcriptome in light and darkness (Tisch et al., 2011b; Tisch and Schmoll, 2013). Mass spectrometry-based proteomics identified the proteins with altered presence or abundance in light and darkness (Table 1).

**Table 1:** Proteins identified by mass spectrometry from QM6a grown in constant light (LL, 1,500 lux) or constant darkness (DD).

**Protein ID**	**Band**	**Predicted size [kDa]**	**Isolated in**	**Gene name**	**Group**	**Family**	**Description**	**Domain annotation**	**Cellulase specific**	**Light regulated**	**Regulation by photoreceptors**					
											**Δenv LL**	***Δenv DD***	**Δblr1 LL**	**Δblr1 DD**	**Δblr2 LL**	**Δblr2 DD**
TR 123456	band A	117	LL		CAZyme	GH 65	candidate acid trehalase	pfam03632, pfam03636, pfam03633	U	NR	NR	NR	NR	NR	NR	NR
TR 64397	band B	78	LL				Neutral/alkaline non-lysosomal ceramidase	pfam 04734	U	NR	U	NR	U	NR	U	NR
TR 51365	band B/J	93	DD LL		Protease		Peptidase S8 family protein, subtilase superfamily, PoS1 related	cd07489; pfam00082, pfam02225, pfam06280	NR	NR	NR	NR	NR	NR	NR	NR
TR 121746	band C	83	LL	*gluc78*	CAZyme	GH 55	candidate b-1,3-exoglucanase; potential pectate lyase; related to GLUC78	pfam12708	U	NR	D	NR	NR	NR	NR	NR
TR 82623	band D	65	LL		Protease		Peptidase S53 family, sedolisin like	cd04056; pfam09286	NR	D	NR	NR	NR	NR	U	U
TR 122780	band D	50	LL	*rgxl*	CAZyme	GH 28	candidate a-glycosidase related to exo-polygalacturonase	pfam00295	U	NR	D	NR	NR	NR	NR	NR
TR 104461	band E	37	LL		CAZyme	CE 1	related to pimeloyl-ACP methyl ester carboxylesterase (COG0596), alpha/beta hydrolase superfamily (cl21494)	COG0596; cl21494; pfam00561	NR	NR	NR	NR	NR	NR	D	NR
TR 111442	band F/R	23	DD LL				distantly related to the ceratoplatanin superfamily	cl06331	D	NR	NR	NR	NR	NR	NR	NR
TR 72183	band G	24	LL				Cyanovrin domain containing protein (pfam08881)	pfam08881	NR	NR	U	NR	D	NR	D	NR
TR 119989	band H	9	LL	*hfb2*			hydrophobin	pfam06766	NR	NR	D	NR	NR	NR	NR	NR
TR 122374	band H	13	LL				unknown	unknown	U	NR	D	NR	NR	NR	NR	NR
TR 49081	band I	87	DD	*cel74a*	CAZyme	GH 74	candidate xyloglucanase; EGL6 (CEL74A)	pfam02012, pfam00734	U	U	D	NR	D	NR	NR	NR
TR 121127	band I/M	87	DD	*bxll*	CAZyme	GH 3	b-xylosidase BXL1	pfam01915, pfam00933	U	U	D	NR	D	NR	NR	NR
TR 72526	band J	93	DD	*glrl*	CAZyme	GH 67	candidate a-glucuronidase GLR1	pfam07488, pfam07477, pfam03648	U	U	D	NR	D	NR	D	U
TR 76672	band K	78	DD	*bgl1/cel3a*	CAZyme	GH 3	b-glucosidase	pfam01915, pfam00933	U	NR	D	NR	D	NR	NR	U
TR 123989	band K	54	DD	*cbh1/cel7a*	CAZyme	GH 7	cellobiohydrolase CBH1	pfam00840, pfam00734	U	NR	D	NR	D	NR	NR	NR
TR 111849	band L	53	DD	*xyn4*	CAZyme	GH 30	xylanase 4	pfam02055	U	NR	D	NR	NR	NR	NR	NR
TR 123026	band N	36	DD				transaldolase/ftuctose-6-phosphate aldolase (IPR001585)	pfam00923	NR	NR	NR	NR	NR	NR	NR	NR
TR 4213	band O	28	DD				ribonuclease T2 (cd01061)	cd01061, pfam00445	U	NR	D	NR	NR	NR	NR	NR
TR 123244	band O	58	DD		Protease		Peptidase S8 family, proteinase K like, subtilase like	pfam05922, pfam00082	U	NR	NR	NR	NR	NR	NR	NR
TR 65406	band P	29	DD		CAZyme	GH 16	candidate b-glycosidase related to cell-wall modifying enzymes	pfam00722	NR	NR	NR	NR	NR	NR	NR	NR
TR 120961	band Q	27	DD	*cel61b*	CAZyme	AA9	lytic polysaccharide monooxygenase, endo-1,4-glucanase; CEL61B	pfam03443	U	NR	D	NR	D	NR	NR	NR
TR 123818	band S	23	DD	*xyn2*	CAZyme	GH 11	xylanase 2	pfam00457	U	U	D	NR	D	NR	D	NR
TR 122127	band T	20	DD				putative cell wall protein related to *A. nidulans* PhiA	unknown	U	NR	D	NR	NR	NR	NR	NR
TR 111915	band U	15	DD				Kazal domain containing protein (IPR011497)	IPR011497	U	D	D	NR	U	NR	NR	NR
TR 108663	band V	14	DD				unknown	unknown	U	NR	D	NR	NR	NR	NR	NR

*Protein IDs refer to the JGI genome annotation of T. reesei (http://genome.jgi.doe.gov/Trire2/Trire2.home.html). Similar band letters indicate that the band contained more than one protein. GH, glycosyl hydrolase; CE, carbohydrate esterase; NR, not regulated. Domain association as determined in the JGI database and with NCBI CDD search is provided. An overview on gene regulation in QM9414 in constant light (LL) or constant darkness (DD) upon growth on cellulose and in photoreceptor mutant strains lacking ENVOY (ENV1), Blue light regulator 1 (BLR1) or Blue Light Regulator 2 (BLR2) is provided. Therefore, data were taken from previous studies (Tisch and Schmoll, 2013; Tisch et al., 2014; Stappler et al., 2017) and are based on fold changes determined by ANOVA analysis of transcriptome abundance with a threshold of p-value 0.01. Detailed data including p-values are provided as Supplementary Material (Data sheet 2). Abbreviations: NR, no regulation; U, upregulation; D, downregulation*.

Several members of different glycoside hydrolase families were found to be secreted in light and in dark conditions, often in clearly different amounts. In light we could identify TR_123456, a candidate a,a-trehalase belonging to the glycoside hydrolase family 65, GLUC78 (TR_121746), a candidate exo-1,3-β-glucanase and member of the glycoside hydrolase family 55 and RGX1 (TR_122780), a candidate polygalacturonase from the glycoside hydrolase family 28. In darkness BXL1 (TR_121127), a β-xylosidase of the glycoside hydrolase family 3, EGL6 (syn. Cel74A; TR_49081), a xyloglucanase belonging to the glycoside hydrolase family 74, GLR1 (TR_72526), a α-glucuronidase of the glycoside hydrolase family 67, BGL1(syn. Cel3A; TR_76672), β-glucosidase 1 belonging to the glycoside hydrolase family 3, CBH1 (syn. Cel7A; TR_123989), the major cellulase in *T. reesei*, member of the glycoside hydrolase family 7, XYN4 (TR_111849), a xylanase of the glycoside hydrolase family 30, TR_65406, member of the glycoside hydrolase family 16 and XYN2 (TR_123818) another xylanase, belonging to the glycoside hydrolase family 11 were found. Additionally, Cel61B (TR_120961), a candidate lytic polysaccharide monooxygenase of glycoside hydrolase family 61, which was re-classified to auxiliary activity family 9 (AA9; Hemsworth et al., 2013) was detected, Moreover, several proteases and other proteins, some with unknown functions were identified (Table 1). Of those proteins, XYN2, BGL1, EGL6, and XYN4 are among the top ranking proteins for limiting hydrolysis capacity of corn stover in *T. reesei* (Lehmann et al., 2016).

We used available microarray data (Tisch and Schmoll, 2013; Stappler et al., 2017) to gain information on light- and carbon dependent regulation of the genes encoding the detected proteins. Most of the genes encoding the identified proteins show upregulation of their transcripts in a cellulase specific manner (Table 1, Data sheet 2). In contrast, only 6 genes of those encoding the 26 identified proteins showed significant light dependent regulation in the microarrays, although the protein pattern showed considerable differences between samples in light and darkness. For example, the gene encoding Cel61B (GH61, band Q) exhibited no significant differences between light and darkness in the microarray, albeit the protein band was only visible in samples from darkness. Also XYN4 (GH30, band L) was only found to be secreted in darkness although no light dependent alteration in transcript abundance was detected (Table 1).

### Light intensity influences the secretome and cellulase activity

**Figure 2:**
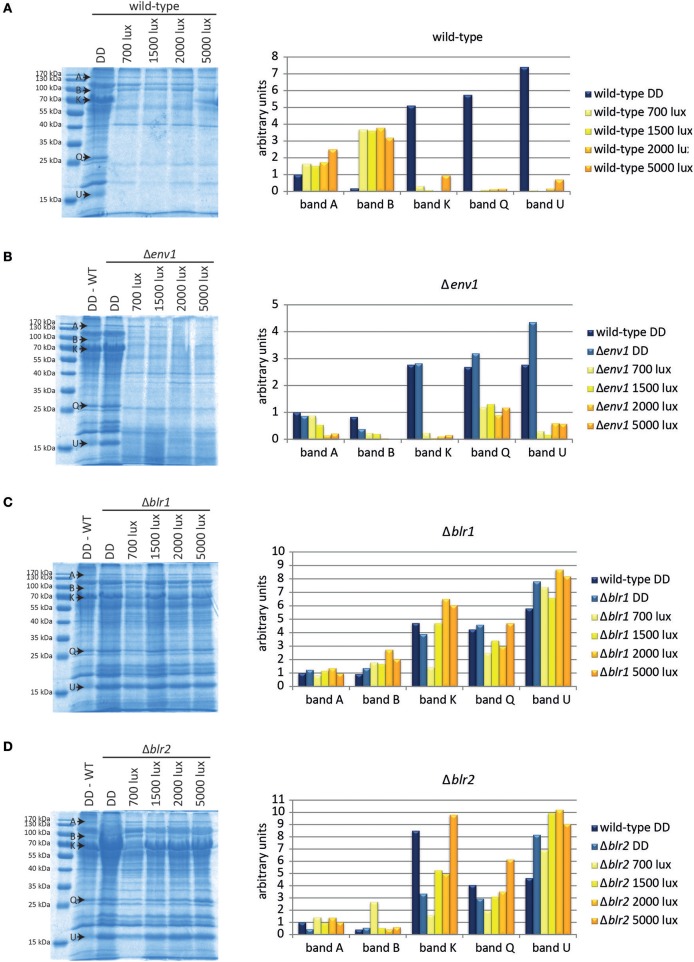
Protein patterns under different light conditions. Proteins from culture filtrate corresponding to equal amounts of biomass of **(A)** the wild-type QM6a, **(B)** Δ*env1*, **(C)** Δ*blr1*, and **(D)** Δ*blr2* grown for 72 h on MA-media supplemented with 1% (w/v) cellulose in constant darkness (DD) or constant light at different light intensities (numbers indicate light intensities in lux) were precipitated and separated by SDS-PAGE. Intensity of 5 selected bands was determined (arbitrary scanning units): band A: GH65, band B: hypothetical ceramidase TR_64397 and hypothetical protease TR_51365, band K: CBH1 and BGL1, band Q: Cel61B (GH61) and band U: putative protease inhibitor TR_111915/CLF1. Replicate SDS-gels are provided in Data sheet 1.

In order to expand our knowledge of light-dependent differences in gene expression and protein production, we examined whether light intensity would result in altered expression patterns or protein abundance. We used four light intensities between 700 lux (low light) and 5,000 lux (high light) and analyzed the protein pattern of secreted proteins by SDS-PAGE. In the wild-type, the striking difference between light and darkness was obvious, but no major differences were seen when the light intensity was varied from 700 to 5,000 lux (Figure 2A).

Five protein bands (A, B, K, Q, and U, Figure 2A) showed particularly interesting responses to light. Band A, identified as TR_123456, a member of the GH65, slightly increased at increasing light intensities. The intensity of protein band B, which contains the hypothetical ceramidase TR_64397 and the hypothetical protease TR_51365, was considerably increased to similar levels in all light conditions compared to darkness. Bands K (BGL1 CBH1), Q (Cel61B), and U (putative protease inhibitor TR_111915) were barely detectable in light-grown cultures, but showed a strong signal in darkness (Figure 2A).

### Deletion of the photoreceptors BLR1 and BLR2 leads to loss of light-specific protein pattern

To investigate the relevance of the photoreceptors ENV1, BLR1, and BLR2 to the light-dependent changes in the secretome pattern, we analyzed secreted proteins of these strains. Protein patterns in Δ*env1* were similar to the wild-type in darkness (Figure 2B). There was a clear difference between proteins secreted under dark conditions and proteins secreted in light in Δ*env1* but no large influence of light intensity. Interestingly, band B, which showed a stronger signal in light in the wild-type QM6a, was decreased in Δ*env1* in light compared to darkness (Figure 2B). In Δ*blr1* and Δ*blr2*, the dark and light patterns were more similar than in the wild type (Figures 2C,D). These results indicate that BLR1 and BLR2 play a major role in regulation of protein abundance in light. The influence of ENV1 is limited to altered regulation of individual proteins, but the overall decrease in protein secretion in light is not dependent on ENV1.

### Cellulase transcription drops to basal levels in high light intensities

**Figure 3:**
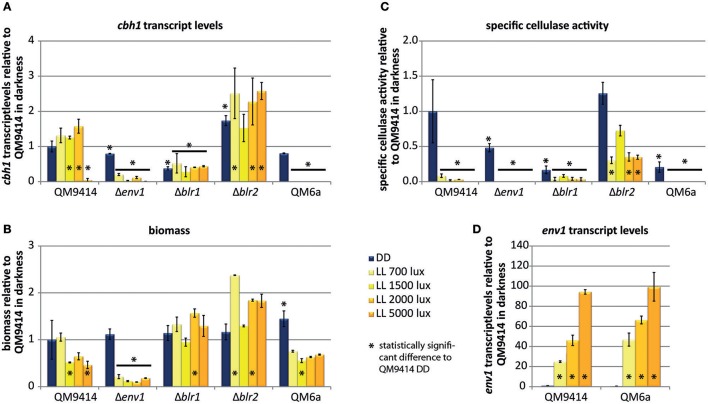
Regulation by different light intensities. **(A)** *cbh1* transcript levels shown relative to QM9414 in constant darkness. **(B)** Biomass shown relative to QM9414 in constant darkness. **(C)** Specific cellulase activity (cellulase activity / biomass) shown relative to QM9414 in constant darkness. **(D)** *env1* transcript levels shown relative to QM9414 in constant darkness. Strains were grown for 72 h on MA-media supplemented with 1% (w/v) cellulose in constant darkness (DD) or constant light (LL) at different light intensities. Five replicates were grown in parallel and used for determination of cellulase activity in the supernatant, which yielded consistent results. Three of the replicate mycelia were used for biomass analysis and two replicates for determination of *cbh1* and *env1* transcript abundance. Errorbars show standard deviations.^*^ indicates values significantly different to QM9414 in darkness (*p* < 0.05). Statistical significance of other comparison is given with *p*-values in the text.

**Figure 4:**
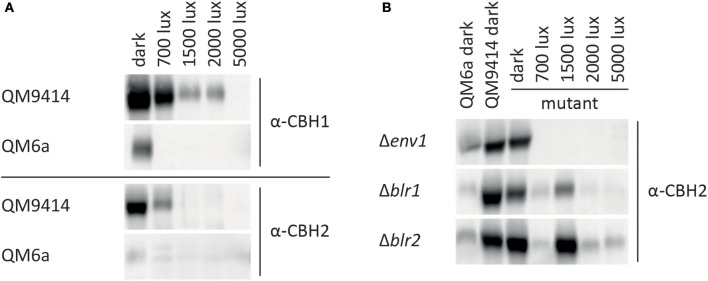
CBH1/Cel7A and CBH2/Cel6A protein levels at different light intensities. CBH1/Cel7A and CBH2/Cel6A in proteins precipitated from culture filtrate corresponding to equal amount of biomass from **(A)** QM6a and QM9414 and **(B)** Δ*env1*, Δ*blr1*, and Δ*blr2* were detected with specific antibodies.

Expression of the major cellulase *cbh1* is influenced by light in *T. reesei* (Schmoll et al., 2005; Castellanos et al., 2010). To investigate the light effect in more detail, we analyzed *cbh1* RNA levels at different light intensities and in photoreceptor-deletion strains. In the wild-type QM9414 *cbh1* transcript levels were increased by up to 50% at light intensities up to 2,000 lux compared to levels in darkness (Figure 3A) in agreement with earlier studies (Schmoll et al., 2005). At a higher light intensity (5,000 lux) *cbh1* levels dropped to basal expression, which is not reflected by biomass production, as the strain did not show a major growth defect under these conditions (Figure 3B). Surprisingly, even though *cbh1* transcript levels were upregulated at moderate light intensities, specific CMCase activity in these samples was severely decreased in QM9414 under all light conditions compared to activity in darkness (Figure 3C). Western blot analysis of culture filtrates revealed that decreased cellobiohydrolase levels were present at higher light intensities (Figure 4A). The signal for CBH1, as well as for CBH2 was the strongest in darkness. At 700 lux less CBH1 and CBH2 was present and the signal decreased further at higher light intensities. At 5000 lux neither CBH1 nor CBH2 was detectable any more. The discrepancy between data for *cbh1* transcript levels, protein abundance and actual activity strongly suggests a level of post-transcriptional regulation of cellulase biosynthesis in light, which is not likely to be limited to *cbh1* and *cbh2*.

### Photoreceptor mutants can still respond to light

Deletion of *env1* leads to decreased *cbh1* transcript levels at all light intensities (*p* < 0.01; Figure 3A), which is in agreement with previous findings for cultivation on cellulose for 72 h (Schmoll et al., 2005; Castellanos et al., 2010). In light Δ*env1* exhibited severely decreased growth (*p* < 0.01; Figure 3B) and cellulase activity was below detection limits due to the low biomass formation (*p* < 0.01; Figure 3C). Moreover, no CBH2 could be detected in culture supernatants from light cultures (Figure 4B). In strains lacking the photoreceptors *blr1* and *blr2, cbh1* transcript levels were not strongly altered by the light condition and they did not show the drop in transcript levels that occurred with the wild-type at the high light intensity of 5,000 lux (Figure 3A). Hence light sensitivity with respect to cellulase transcription is alleviated in Δ*blr1* and Δ*blr2*. Expression levels of *cbh1* in Δ*blr1* were generally lower than in the wild-type but stayed at the same level independent of the light condition. In Δ*blr2, cbh1* levels were increased compared to the wild-type at high light conditions (5,000 lux; *p* < 0.01) and thereby largely resembled the increase of transcript levels seen in the wild-type, albeit this increase was only reached at 5,000 lux instead of 2,000 lux in the wild-type (Figure 3A). In contrast to transcription data, both Δ*blr1* and Δ*blr2* exhibited decreased specific cellulase activity in light compared to darkness, irrespective of the light intensity (*p* < 0.01; Figure 3C). Nevertheless, cellulase levels in Δ*blr2* in light were still higher than in the wildtype under the same conditions (*p* < 0.01). Detection of cellulase abundance in western blot analysis was in agreement with these results (Figure 4B). Consequently, strains lacking one of the photoreceptors were still responsive to light and influenced cellulase gene expression, hence confirming earlier results (Castellanos et al., 2010; Gyalai-Korpos et al., 2010; Schmoll et al., 2012; Tisch and Schmoll, 2013).

### QM9414 is more light tolerant than QM6a

QM9414 represents a derivative of the natural isolate of *T. reesei* QM6a with improved cellulase gene expression (Vitikainen et al., 2010; Seiboth et al., 2011). We investigated the difference in sensitivity to light of these two strains. In QM6a *cbh1* transcript levels in light were dramatically downregulated and hardly detectable at all light intensities whereas in QM9414 downregulation of *cbh1* occured only at the highest tested light intensity of 5,000 lux (Figure 3A), even though comparable amounts of biomass are produced in the two strains at 1,500–5,000 lux (Figure 3B). In darkness transcript levels of *cbh1* showed only a slightly negative trend in QM6a compared to QM9414 (Figure 3A), but cellulase activity levels were considerably lower in QM6a (Figure 3C). Data for specific cellulase activity of QM6a in light however, showed no detectable activity and western blotting did not reveal presence of CBH1 or CBH2 in light, whereas in QM9414 both were present at least for low to moderate light intensities (Figures 3C, 4A). Also, the coregulation of CBH1 and CBH2 was not broken by light, independent of the intensities. This indicates that light tolerance with respect to cellulase production of the QM6a derivative QM9414 is improved compared to its parental strain. It remains to be investigated, whether light tolerance generally correlates with improved enzyme production.

### Transcript levels of *env1* increase with rising light intensity

It was shown previously that *env1* transcript levels are upregulated in response to light (Schmoll et al., 2005). Here we show that light intensity directly affects the magnitude of *env1* upregulation also in *T. reesei* (Figure 3D). In QM9414 *env1* transcript levels were about 25 times higher at 700 lux than in darkness. With increasing light intensity also transcript levels increased until up to 94-fold upregulation at 5,000 lux. In the more light sensitive strain QM6a *env1* transcripts are already 88 fold upregulated at 700 lux compared to its levels in darkness. At 5,000 lux *env1* is expressed 186 times more than in darkness. In darkness *env1* transcript levels show a negative trend in QM6a compared to QM9414. In the photoreceptor deletion strain Δ*blr1* transcript levels of *env1* are not influenced by light anymore and remain at the low darkness levels when exposed to light. Lack of *blr2* results in consistently decreased transcript abundance of *env1* in light and darkness (*p* < 0.01) compared to dark levels in QM9414 (data not shown). These findings are in agreement with earlier studies (Castellanos et al., 2010) showing that the presence of BLR1 and BLR2 is necessary to activate *env1* transcription upon exposure to light. Additionally, the positive regulation of *env1* in light is in agreement with a positive effect on cellulase transcription in light (Figures 3A,D).

### Deletion of *clf1* leads to decreased protease activity

**Figure 5:**
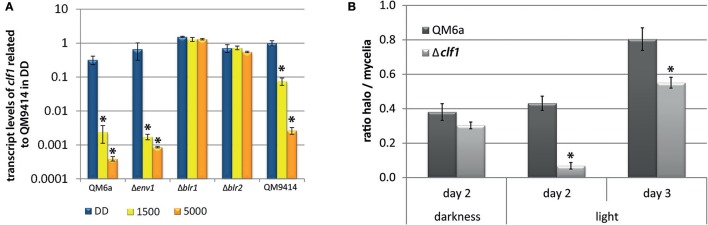
Transcriptional regulation of *clf1* and protease activity in Δ*clf1*. **(A)** Transcript abundance of clf1 in constant darkness (DD) or in constant light with 1,500 lux (“1,500”) or 5,000 lux (“5,000”). Strains were grown for 72 h on MA-media supplemented with 1% (w/v) cellulose in constant darkness (DD) or constant light at different light intensities. Two replicates for determination of *cbh1* and *env1* transcript abundance. **(B)** For determination of protease activity, strains were grown on milk TSA agar at 28°C in constant darkness (DD) or constant light (LL). Halo formation by proteases and hyphal extension was determined at the indicated time points. Halo diameter was related to the respective diameter of the mycelium under the respective condition. Ratio of halo to mycelia is given for the wild-type QM6a (dark gray) and Δ*clf1* (light gray). Three biological replicates and two technical replicates were used. Errorbars show standard deviations.^*^ indicates values significantly different to QM9414 in darkness (*p* < 0.05). Statistical significance of other comparison is given with *p*-values in the text.

Analysis of the secretome in light and darkness revealed several interesting proteins. One band clearly visible in dark-grown cultures, but absent in light, was band U (Figure 1). We identified this band by mass spectrometry as TR_111915. This gene shares homology with protease inhibitors that contain Kazal domains (Interpro domain IPR011497) (*e*-value 7e-48). Transcriptome data showed that TR_111915 transcript levels were increased upon growth on cellulase-inducing carbon sources like cellulose, lactose or sophorose (Stappler et al., 2017). Furthermore, TR_111915 was downregulated in response to light and was a target of ENV1, as well as of the adenylate cyclase (ACY1) in light (Schuster et al., 2012b; Tisch and Schmoll, 2013; Tisch et al., 2014). We tested the regulation of *clf1* upon growth on cellulose in darkness and in light of 1,500 and 5,000 lux by RTqPCR. This analysis confirmed the downregulation in light with a more severe effect with the higher light intensity (Figure 5A). In QM6a, transcript abundance was below that of QM9414 under all conditions. In strains lacking BLR1 or BLR2, dark levels were around those in the wild-type and did not decrease in light (Figure 5A). As these data point at a light- and photoreceptor dependent relevance of TR_111915 for enzyme abundance, we designated this gene *clf1* (cellulase and light associated *f* actor 1) and prepared a knock-out strain of this gene in the QM6aΔ*ku80* background.

The phenotype of Δ*clf1* did not show growth or sporulation defects. Since CLF1 contains a putative protease inhibitor domain we analyzed protease activity. In cultures grown in light, Δ*clf1* secreted protease activity was significantly decreased compared to the wild-type. Also in darkness protease activity was decreased, but to a smaller extent than in light. (Figure 5B). Consequently, CLF1 has an influence on protease activity, but it is not a protease inhibitor, rather it is a protein with positive effect on their activity and hence is not responsible for the difference in enzyme/protein abundance between light and darkness. Analysis of transcript levels of *cbh1* and specific cellulase activity in liquid cultures of the wild-type QM6a, QM6a Δ*ku80*, and Δ*clf1* grown in constant light and constant darkness on MA media with cellulose as carbon source showed no significant differences between the deletion strain and the wild-type (data not shown). Hence, CLF1 is not involved in regulation of cellulase gene expression or potential degradation of secreted cellulases in light.

## Discussion

In this study we investigated the effect of light on the secretome of *T. reesei*. Light strongly influences the composition of the secretome in that the amount of secreted proteins decreases considerably in the wild-type and differential regulation in light and darkness was observed. Additionally, light intensity influences the secretome, albeit the effect is minor compared to the difference in protein abundance between light and darkness.

We found several members of different glycoside hydrolase families to be differentially regulated in light and in dark conditions. Many of them show different intensities of the band on the SDS-PAGE-gel, indicating that their amount present in the media differs depending on the light condition. These results are in agreement with previous studies showing that glycoside hydrolases are an important target of light signaling (Tisch et al., 2011b; Tisch and Schmoll, 2013). Also proteins with other function, for instance proteases or proteins with unknown functions, were present in different amounts after growth in constant light or constant darkness. One of the proteins identified from the culture grown in light was the hydrophobin HFB2. It has been shown that *hfb2* is regulated by the G-alpha protein GNA1 and in response to light (Nakari-Setälä et al., 1997; Seibel et al., 2009). Interestingly, *hfb2* transcript levels are downregulated in light on lactose, sophorose and glycerol, but not on cellulose. Furthermore, *hfb2* shows regulation by cellulase-inducing conditions only in darkness, not in light (Stappler et al., 2017). Moreover, *hfb2* is involved in sporulation (Askolin et al., 2005) and it is highly expressed upon growth in media containing complex plant polysaccharides, cellulose, xylan, cellobiose, or lactose, as well as in response to N and C starvation (Nakari-Setälä et al., 1997). The lower amount of plant cell wall degrading enzymes secreted in light may explain the presence of HFB2 in the secretome due to an effect of starvation.

Surprisingly, microarray data from previous studies (Tisch and Schmoll, 2013) revealed that for several proteins which showed different secretion levels in light and in darkness, the corresponding genes are not regulated at the transcriptional level in response to light. Although it was previously reported that genes of predicted secreted proteins have a positive correlation to the extracellular specific protein production rate (Arvas et al., 2011), our data indicates that in addition to the transcriptional regulation, regulation at another level occurs. This is in agreement with other studies which show that in *T. reesei* a strict correlation of transcription and secretion of proteins is not always present and that already 2 min after induction changes in the phosphoproteomic profile can be detected that indicate a complex regulation pathway (Schuster et al., 2011; Nguyen et al., 2016). Also in *N. crassa* extensive post-transcriptional regulation was reported (Xiong et al., 2014). The pathway from gene transcription to protein secretion contains many steps, all of which are potential targets for additional regulation (for a review see Conesa et al., 2001). Recently, we could show, that a G-protein coupled receptor (CSG1) impacts post-transcriptional regulation of cellulase gene expression. In the absence of CSG1, cellulase transcript levels remain almost unaltered, while secreted cellulase activity drops dramatically on cellulose and lactose (Stappler et al., 2017). Consequently, cellulase regulation comprises a transcriptional and a post-transcriptional section, for which we found support also in this study.

Analysis of *cbh1* transcript levels and the specific cellulase activity indicate as well that another regulation step is present. Transcript levels of *cbh1* increase in light whereas the specific cellulase activity is dramatically decreased in light in QM9414. A similar discrepancy can be seen for the *blr1* and *blr2* deletion strains. Transcript levels of *cbh1* are not regulated in response to light, but specific cellulase activity is strongly decreased in light. Although transcript levels of only one cellulase were determined and cumulative activity of all cellulases was measured, a correlation between the results would be expected, as it has been postulated previously that cellulases are transcriptionally co-regulated (Ilmen et al., 1997; Foreman et al., 2003).

We showed that the presence of light is an important signal for *T. reesei* but also that the intensity of light is relevant. Even though differences between light and darkness are of a much bigger magnitude, the intensity of light still affects expression of genes. ENV1, one of the photoreceptors, has been shown to be necessary for photoadaptation and for the response to increased light intensities (Schmoll et al., 2005; Schuster et al., 2007; Castellanos et al., 2010; Tisch et al., 2011b). Our study confirms that it clearly responds to different levels of light. Transcript levels of *env1* are strongly upregulated even at a low light intensity of 700 lux. With increased light intensity, also *env1* transcript levels increase almost proportionally to the intensity.

In our study we analyzed both the original isolate of *T. reesei* QM6a, as well as its derivative, the enhanced cellulase producer QM9414. Our results show some interesting differences between these two strains in respect to light sensitivity. In QM6a in light, transcription of *cbh1* is strongly decreased compared to levels in darkness and only at basal levels. In contrast, in QM9414 *cbh1* levels increase at low to moderate light intensities, but at 5,000 lux *cbh1* transcript levels drop dramatically. These results indicate that *T. reesei* in nature upon encounter of light decreases its cellulase production. The laboratory strain QM9414 was mutagenized to obtain a mutant that produces high levels of cellulase (Mandels et al., 1971). As a result it seems to also be less sensitive to light. Genomic alterations between QM6a and QM9414 were analyzed by Vitikainen et al. (2010) by comparative genomic hybridization analysis, but no mutations that could explain the differences in light sensitivity were found. As mentioned above, transcript levels of *env1* increase with rising light intensity. At the lowest tested light intensity of 700 lux, QM6a *env1* transcript levels are almost twice as high as in QM9414. Much stronger light (2000 lux) is needed for QM9414 to reach this level. As ENV1 is involved in adaptation to light, it seems that the more light-tolerant strain QM9414 upregulates *env1* less than the more light-sensitive strain QM6a.

Analysis of the secreted proteins revealed an interesting unknown protein, TR_111915. The band containing this protein (Figure 1, band U) is clearly present upon growth in darkness, but absent in light conditions. It is homologous to known protease inhibitors containing Kazal domains (Interpro domain IPR011497), which are known to specifically inhibit S1 serine proteases (Schmoll et al., 2016). TR_111915 is one of only two putative proteinase inhibitors found in *T. reesei* (Schmoll et al., 2016). For industrial production of enzymes the presence of naturally produced proteases by the fungus often constitutes a problem, as they can degrade the desired product and diminish production rates and enzyme stability. Therefore, proteases are often missing in commercial enzyme preparation due to selection against proteases or targeted deletion (Nagendran et al., 2009). Due to this relevance of proteases in industry the presence of a potential protease inhibitor, that is regulated in response to cellulase inducing condition as well as to light and ENV1 (Tisch and Schmoll, 2013; Tisch et al., 2014; Stappler et al., 2017), was of interest. To test the function of TR_111915 a deletion strain was constructed. Even though TR_111915 shares homology with protease inhibitors our data indicates that it in *T. reesei* it serves a different function. Contrary to expectations, deletion of this gene leads to decreased protease activity under the tested conditions, indicating a not yet detected role and possibly an involvement in enhancing protease activity.

In summary, we show that light influences the secretome of *T. reesei* and that its regulation takes place not only at the transcriptional level, which is in agreement with recent studies. We found that the original isolate of *T. reesei* QM6a is more light sensitive than its derivative QM9414. These results show the importance of controlled light conditions for optimization of industrial strains. Even though they might not be as light sensitive as the original isolate QM6a anymore, they still clearly respond to light as do currently applied industrial production strains (unpublished results). Additionally, the light intensity should be taken into account since also in the light tolerant strain QM9414 a strong reaction and drop in *cbh1* transcription was present at high light intensity.

## Author contributions

ES performed light response experiments with *T. reesei*, strain construction and analysis and drafted the paper, JW performed mass spectrometric analysis and participated in drafting the manuscript, SB contributed to statistical analysis and MS conceived the study and wrote the final version of the manuscript.

### Conflict of interest statement

The authors declare that the research was conducted in the absence of any commercial or financial relationships that could be construed as a potential conflict of interest.
