# Supplementary material for: Abundance of Secreted Proteins of Trichoderma reesei Is Regulated by Light of Different Intensities
Source: Front Microbiol. 2017 Dec 22;8:2586. doi: 10.3389/fmicb.2017.02586 (PMC5770571; doi:10.3389/fmicb.2017.02586)
Supplement: Supplementary file 1 [file DataSheet1.PDF]

Abundance of secreted proteins of *Trichoderma reesei* is regulated by light of different intensities

Eva Stappler[1], Jonathan D. Walton[2], Sabrina Beier[1] and Monika Schmoll[1]*
[1] AIT - Austrian Institute of Technology GmbH, Department Health and Environment, Bioresources, Konrad-Lorenz-Straße 24, 3430 Tulln, AUSTRIA
[2]DOE Great Lakes Bioenergy Research Center, DOE Plant Research Laboratory, and Department of Plant Biology, Michigan State University, East Lansing, MI 48824, USA

SUPPLEMENTARY MATERIAL

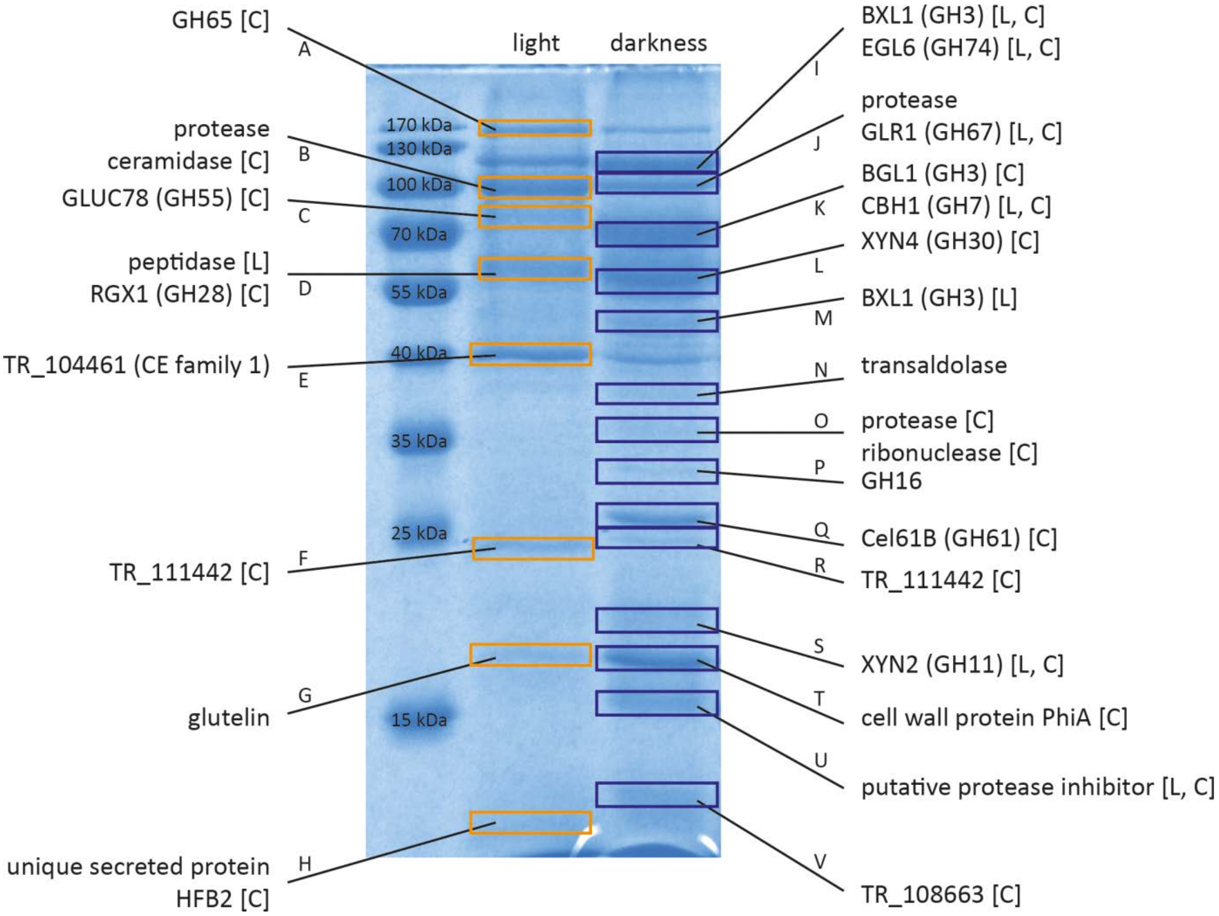

Figure S1: **Proteins identified by mass spectrometry**. Boxes indicate the area excised from the gel for mass spectrometry analysis. Proteins were separated by SDS-PAGE and selected bands were excised and subjected to LC/MS/MS. Proteins were identified using the Mascot searching algorithm. [L] indicates regulation of the corresponding gene in response to light at the transcriptional level [C] indicates regulation of the corresponding gene under cellulase-inducing conditions.
QM6a was grown on Mandels Andreotti minimal medium with 1 % (w/v) cellulose as carbon source for 72 hours in constant darkness or constant light (1500 lux).

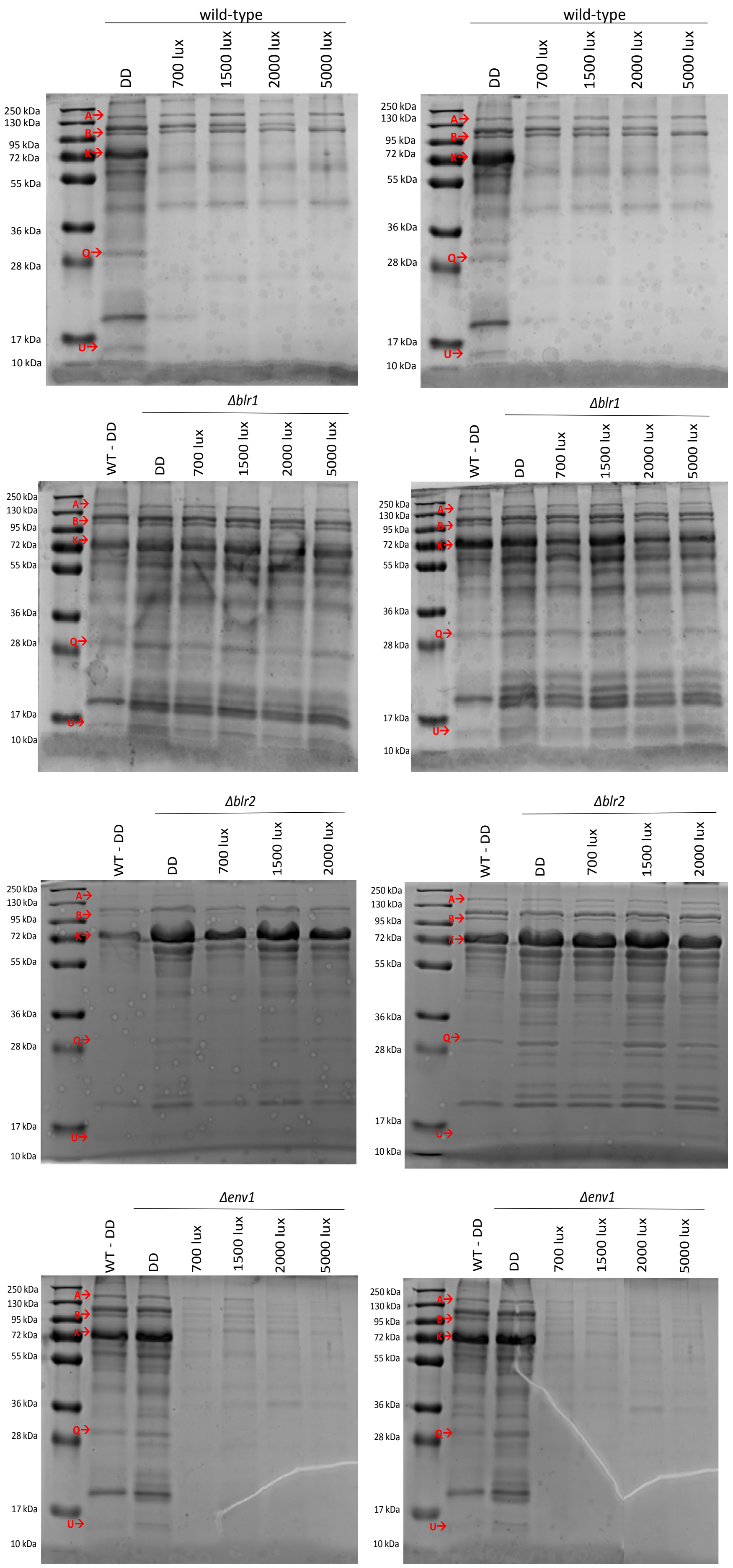

Figure S2. **Replicate gels to those shown in Figure 2.**
